# ZnO Nanostructure Templates as a Cost-Efficient Mass-Producible Route for the Development of Cellular Networks

**DOI:** 10.3390/ma9040256

**Published:** 2016-03-31

**Authors:** Eleni Makarona, Beatrix Peter, Inna Szekacs, Christos Tsamis, Robert Horvath

**Affiliations:** 1 Institute of Nanoscience and Nanotechnology, NCSR “Demokritos”, Aghia Paraskevi, Athens 153 10, Greece; c.tsamis@inn.demokritos.gr; 2 Nanobiosensorics Momentum Group, Institute of Technical Physics and Materials Science, Centre for Energy Research, Budapest 1121, Hungary; pbeatrix@mfa.kfki.hu (B.P.); inna.szekacs@gmail.com (I.S.); horvathr@mfa.kfki.hu (R.H.); 3 Doctoral School of Molecular- and Nanotechnologies, University of Pannonia, Veszprém 8200, Hungary

**Keywords:** ZnO nanostructures, HeLa cells, selective adhesion, engineered cellular networks, nanotopography

## Abstract

The development of artificial surfaces which can regulate or trigger specific functions of living cells, and which are capable of inducing *in vivo*-like cell behaviors under *in vitro* conditions has been a long-sought goal over the past twenty years. In this work, an alternative, facile and cost-efficient method for mass-producible cellular templates is presented. The proposed methodology consists of a cost-efficient, two-step, all-wet technique capable of producing ZnO-based nanostructures on predefined patterns on a variety of substrates. ZnO—apart from the fact that it is a biocompatible material—was chosen because of its multifunctional nature which has rendered it a versatile material employed in a wide range of applications. Si, Si_3_N_4_, emulated microelectrode arrays and conventional glass cover slips were patterned at the micrometer scale and the patterns were filled with ZnO nanostructures. Using HeLa cells, we demonstrated that the fabricated nanotopographical features could promote guided cellular adhesion on the pre-defined micron-scale patterns only through nanomechanical cues without the need for further surface activation or modification. The basic steps of the micro/nanofabrication are presented and the results from the cell adhesion experiments are discussed, showing the potential of the suggested methodology for creating low-cost templates for engineered cellular networks.

## 1. Introduction

*In vitro* cellular studies and analysis have become powerful tools in the hands of biology, drug discovery and our understanding of disease prevention, prognosis, and diagnosis. Nonetheless, standard culturing procedures in culture flasks, petri dishes or microwell plates cannot fully replicate the *in vivo* conditions occurring around living cells. In addition, observing cells within pieces of tissues, or even primary and secondary cell cultures does not allow to fully comprehend the underlying mechanisms of cellular network formation, connectivity, signaling pathways, and cell inter-dependencies to be elucidated. Over the past twenty years, intense research efforts have focused on the development of man-made, artificial surfaces which can regulate or trigger specific functions of living cells, and which are capable of inducing *in vivo*-like cell behaviors under *in vitro* conditions. One major goal of this cumulative research effort was to produce templates or scaffolds where cells would be “manipulated” to occupy specific locations and then be guided to form connections over predefined pathways. Such templates would enhance several biomedical fields covering the entire spectrum from fundamental cellular biology studies to cell-based biosensors for drug development [1,2], tissue engineering, and regenerative medicine [3]. Some characteristic examples which show the vast potential of cellular templates and scaffolds are the following: improving neurophysiological studies through the use of microelectrode arrays (MEAs), where each neuronal cell would be guided on top of a recording/stimulating electrode, while at the same time connected with other neuronal cells sitting on top of a matrix of electrodes, and their interconnectivity could be recorded (this is still an open issue) [4,5,6]; pharmacological studies via cellular networks of controlled topography and interconnectivity [7], and/or co-cultures [8]; cell-based biosensors and cell-on-chip applications, where the cells either play the role of the transducer itself [9,10] or remain the object under investigation [11,12]; cellular self-repair [13] or artificial generation of organs, bone tendons, ligaments, cartilage or even intervertebral discs to replace damaged parts without the need for transplants or in cases where transplants are not possible.

At the same time, micro- and-nanotechnology emerged as a valuable ally towards the realization of the above-mentioned goals. Micro- and- nanofabrication techniques routinely used for MOEMS/NOEMS started to be employed as new platforms for biological studies, and it soon became apparent that electronic devices may as well serve as bioanalytical tools and not just as building blocks of electronic circuitry. Initially, the problem was approached from a chemistry point-of-view, because environmental sensing by cells involves specific binding between cellular receptors and extra-cellular matrix (ECM) ligands. During the late 1990s and early 2000s, several approaches were suggested, mainly involving photolithographic patterning and modification of surfaces—some characteristic, but not exhaustive examples can be found in [8,14,15,16]—or microcontact printing and stencil techniques as in e.g. [17,18].

However, later studies began to reveal that not only surface chemistry and surface modification schemes, but also the micro/nanotopographical features of the substrates used for cell culturing, play a pivotal role in cell viability, proliferation, migration, and functionality (characteristic examples can be found in the literature [19,20,21,22]). The reason behind such a regulatory behavior mediated by purely mechanical cues (such as roughness, rigidity/elasticity, anisotropy *etc.*) is that nanometer-scale objects are physiologically relevant to the focal adhesions and the ECM matrix (5–200 nm) [23,24]. As suggested in [23] there exists a critical length between 58 nm and 73 nm for the separation of integrins crucial for focal contact formation, therefore the particular morphological characteristics of nanostructured substrates may seem to play a critical role on cellular attachment and behavior irrespective of any chemical surface modification. This of course does not exclude combined physicochemical effects on cell attachment, e.g. [25] or [26], but may offer a new means for simplifying substrate preparation for cellular cultures.

In this work, an alternative methodology for the realization of cost-efficient templates is introduced. The fabricated substrates for cell seeding are comprised of μm-size patterns containing ZnO nanostructures. We will show that the fabricated purely nanotopographical cues can promote selective cellular adhesion on predefined patterns. The proposed methodology is fully compatible with mainstream microfabrication techniques, and has thus the potential for fast laboratory-to-market times and easy transfer to mass-production. It has at its core the hydrothermal growth of ZnO nanostructures, which is a very versatile method allowing the control of the morphological characteristics of the nanostructures through tuning of simple key environmental parameters [27,28,29]. In addition, this method is facile, rapid, of extremely low cost and can be applied to a variety of substrates ranging from conventional Si wafers to flexible substrates such as Kapton [30], PDMS (poly(dimethylsiloxane)) [31], and PET (polyethylene terephthalate) [32]) as well as less conventional materials such as wood [33], paper [34], and textiles [35].

Apart from the ease-of-use of the method itself, the choice of material is based on the fact that ZnO is a multi-faceted semiconductor donned with a unique combination of physical properties [36] that render it a versatile material employed in a plethora of industrial branches [37,38]. In particular, ZnO nanostructures that can be produced via several methods [37,39,40,41,42,43] and whose morphology plays a crucial role in their functionality, have found applications in a wide variety of devices. This wide spectrum of ZnO nanostructure-applications spans to include UV sensors [44], gas sensors [45], photovoltaics (dye sensitized solar cells) [46,47], inverted bulk-heterojunction solar cells [48,49], thin-film solar cells [50], optoelectronic devices [40,51] as well as energy harvesting devices, such as piezoelectric [52] or triboelectric [53] energy harvesters, or even energy storage systems, such as electrochemical supercapacitors [54] or Li-ion batteries [55]. Recently, it was established that ZnO nanostructures can support mammalian cellular growth and promote selective cellular adhesion for specific cell lines [56,57,58], while at the same time exhibiting prophylactic and therapeutic effects against viruses without affecting the viability of mammalian cells [59,60] . Therefore, it seems that ZnO nanostructure templates may offer a suitable route to promote selective cellular adhesion and serve as suitable templates for cellular networks. Given the fact that ZnO nanostructures can also become the functional part of optoelectronic devices, one could envisage that the nanostructures may play a dual role guiding the cells to specific locations via nanotopographical cues and be themselves the functioning electrode or light-emitting device probing the adhered cells.

This work focused on developing ZnO nanostructure-based templates of various patterns on Si, Si_3_N_4_, metallic (Al and Pt) substrates as well as on conventional glass cover slips routinely used in optical microscopy to explore the potential of the proposed methodology for cellular templates. The applications of the fabricated structures could expand to electronic devices, optical biosensors, microelectrode arrays, or transparent templates for real-time observations of live cell status and behavior. As a study case, HeLa cell line was chosen, because it is the most commonly used adherent human carcinoma cell line in research and development for understanding many fundamental biological processes. The cells were cultured on all templates up to 4 days and were observed with optical and scanning electron microscopy (SEM, JEOL JSM -7401f, Tokyo, Japan) after 2 days and 4 days in culture in an effort to elucidate whether the nanotopographical features can promote guided cellular adhesion on pre-defined patterns.

## 2. Results

### 2.1. HeLa Cultures on Patterned Si Wafers with ZnO Nanostructures

The HeLa cells were cultured on Si wafers with photolithographically defined patterns containing ZnO nanorods (see Section 4 for details) over the course of 4 days in order to explore whether this cell line can adhere preferentially onto the predefined patterns. Half of the cultures were observed with SEM and optical microscopy on day 2 and the remaining ones on day 4. Bare Si substrates and conventional cover slips were used as references. The patterned Si wafers were identical to the templates described in detail in [57] which had already been tested with another cell line, namely Neuro2A, in order to establish whether the same nanotopography has the same effect on different cell lines. Briefly, in [57] it was observed that Neuro2A cells tend to preferentially adhere onto the areas containing nanorods and only a small fraction of the population remains on the flat areas. The highest proliferation rates were observed on patterns containing densely packed vertically-aligned nanorods, while the lowest proliferation rates were observed on patterns were the nanorods were grown at larger angles despite the fact that the average nanorod diameter was the same. This decrease was attributed to the fact that larger angles result in larger spacings between the nanorods and a decreased total effective area where the neuronal cells could form stable adhesion focal points. Note, these findings are in agreement with the observations of Spatz and co-workers [23] who showed that the focal adhesion assembly requires the spacing between ligand integrins to be less than 70 nm. Spacing larger than 73 nm between ligated integrins limits attachment, spreading, and actin stress fiber formation.

**Figure 1:**
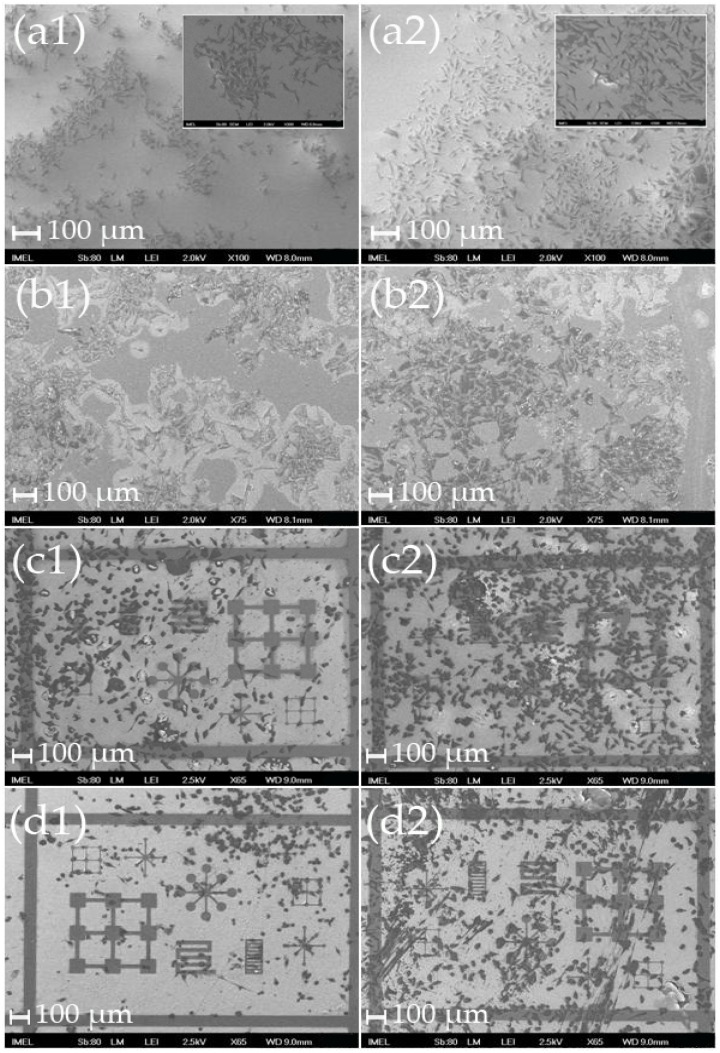
Scanning electron microscopy (SEM) images of HeLa cultures on (**a**) cover slips (control 1); (**b**) bare Si wafers (control 2); (**c**) “Neuro2A less-friendly” patterned Si with ZnO nanostructures; and (**d**) “Neuro2A most-friendly” patterned Si with ZnO nanostructures after 2 days (left column) and 4 days (right column). Scale bar: 100 μm. Insets of (**a1**) and (**a2)**: Higher magnification images of the cell cultures on the cover slips to show the shapes of the cells (scale bar of insets: 10μm).

Results from the HeLa cultures are shown in Figure 1, while the main observations are the following: (a) The cells that adhered on the ZnO nanostructures were smaller in size and more round compared to the cells on bare silicon and on the cover slips (controls); (b) the HeLa cells had the opposite behavior to Nuero2A: it seems that the proliferation rate was decreased on samples that had favored the high proliferation rates of Neuro2A, while the proliferation rate on “Neuro2A-less friendly” samples was higher (see Figure 1(c1) and Figure 1(c2) versus Figure 1(d1) and Figure 1(d2)); (c) It was not possible to determine with certainty whether the HeLa cells adhere preferentially onto the nanostructures or the flat surfaces. The ambiguity mainly stemmed from two reasons. Firstly, the HeLa cells are much larger than the patterns of the samples, therefore even in the case where the cells would have preferred to adhere onto the flat surfaces, there was not enough room to accommodate their somata within the patterns. Secondly, the HeLa cells are very resilient cells and have in general high proliferation rates, therefore on day 4 the cells had covered the entire sample, so it was not possible to discern any preference on adhesion sites (see Figure 1(c2)). Still, it seems that HeLa, contrary to Neuro2A, tend to avoid the nanostructured areas.

**Figure 2:**
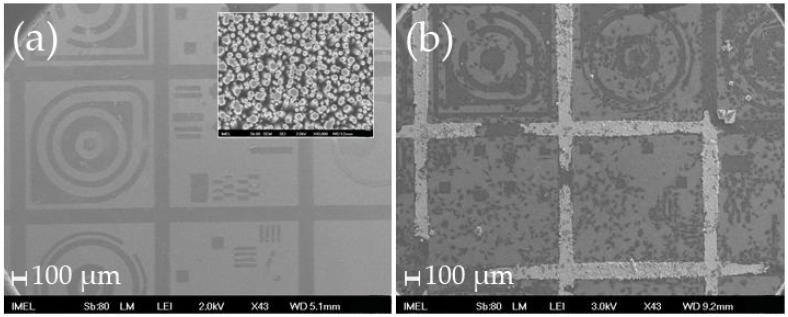
SEM images of (**a**) of the patterned Si wafer with larger patterns and larger flat areas (the darker areas contain the ZnO nanorods shown in the zoom-in inset image; scale bar: 100 nm); (**b**) the same samples with HeLa cells after 4 days in culture, where one can see that the cells mostly adhere onto the flat areas (the white areas are the nanorods that have been covered by salts from the nutrient medium that was not fully removed after washing). Scale bar: 100 μm.

For that reason, the experiments were repeated with other types of patterns, which were not only larger, but had larger flat surfaces compared to the samples used for the Neuro2A cultures and in the first part of this work (hereafter referred to as samples “L” to distinguish them from the samples of smaller patterns hereafter referred to as samples “S”). As seen in Figure 2, when larger patterns were employed, the HeLa cells tended to move towards the flat surfaces and avoided the nanostructures as evidenced by their somata that conformed to the shapes of the patterns.

### 2.2. HeLa Cultures on Si_3_N_4_

Within this work, effort was also put in to create cellular networks on materials that are commonly used for optical biosensing applications and more specifically in label-free optical biosensors. One of the most common materials used for waveguides is silicon nitride (Si_3_N_4_). Towards that purpose Si_3_N_4_ layers were deposited onto conventional 3”- and 4”-Si wafers and subsequently were patterned and modified with ZnO nanostructures in a similar way to the one employed for Si wafers (for details please refer to Section 4). The cultures followed the same protocols as the ones used for the Si substrates. Both types of patterns, smaller and larger ones were used in an analogous way to the Si substrates.

**Figure 3:**
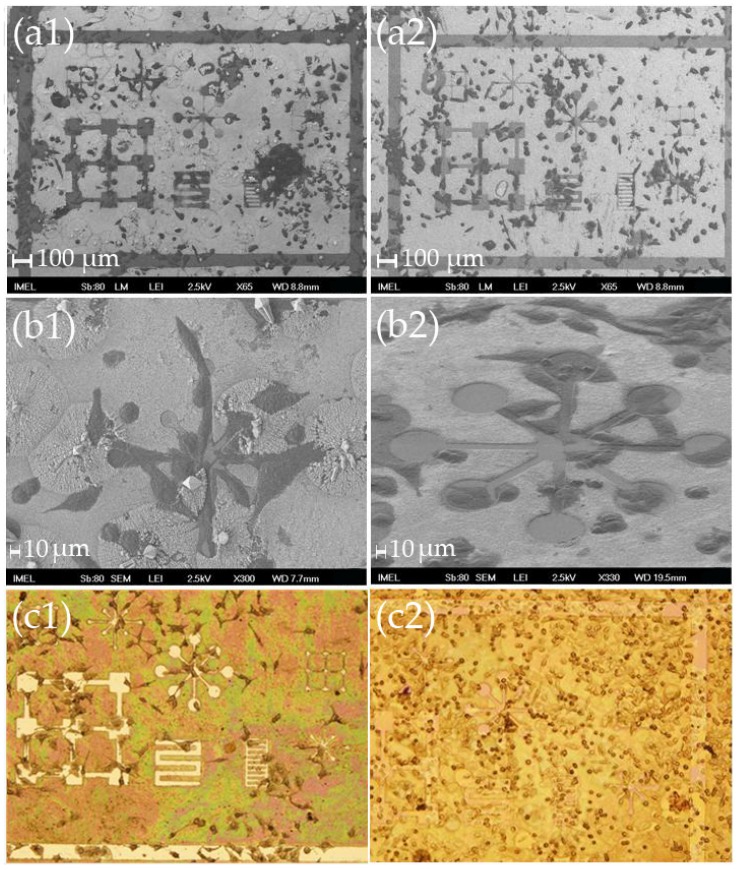
(**a**); (**b**) SEM images at two different magnifications (scale bars: 100 μm and 10 μm, respectively) and (**c**) optical microscope images of HeLa cells after 2 days (left column), and 4 days (right column) in culture on modified nitride samples “S” with small free patterns, where it can be seen that despite the limited flat surface the cells tend to avoid the nanostructured areas and try to “squeeze” inside the patterns. On day 4, the cells that adhered onto the nanostructures were no longer elongated and obtained a more spherical shape. (**a1**); (**b1**); and (**c1**) were obtained after 2 days in culture, while (**a2**); (**b2**); and (**c2**) were obtained after 4 days in culture.

As seen in Figure 3, the HeLa cells preferentially adhered onto the flat nitride surfaces as soon as day 2. They appeared to conform to the shape of the patterns and to adhere onto the borderlines. Even in the case of samples “S” of limited free areas, the cells seem to try to squeeze inside the patterns so as to avoid the nanostructured surfaces. In addition, on day 4 the cells that were forced to sit on the nanostructured areas because of the fast proliferation rates, obtained a more spherical shape than their usual elongated one (see Figure 3(b2) and Figure 3(c2)). 

**Figure 4:**
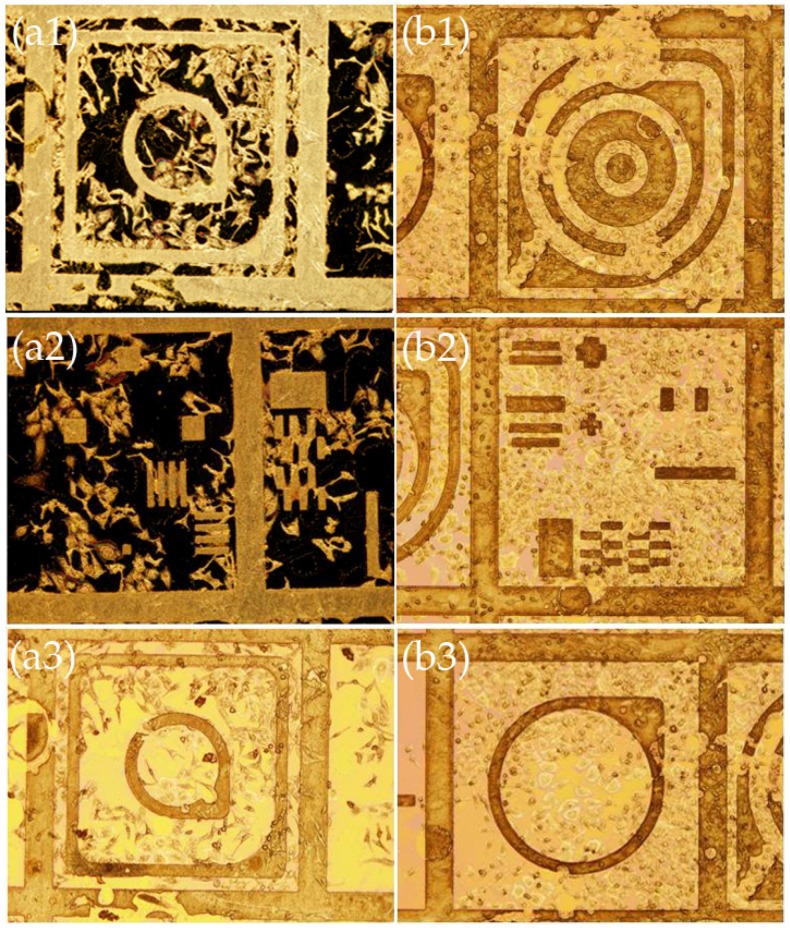
Optical microscope images of HeLa cells on various patterns after (**a**) 2 days; and (**b**) 4 days in culture on modified nitride samples “L” with large patterns and large flat areas showing the preference of the cells to adhere onto the flat surfaces avoiding the nanostructures. (**a1**); (**a2**); and (**a3**) were obtained at several locations of the template after 2 days in culture. (**b1**); (**b2**); and (**b3**) were obtained at several locations of the template after 4 days in culture.

**Figure 5:**
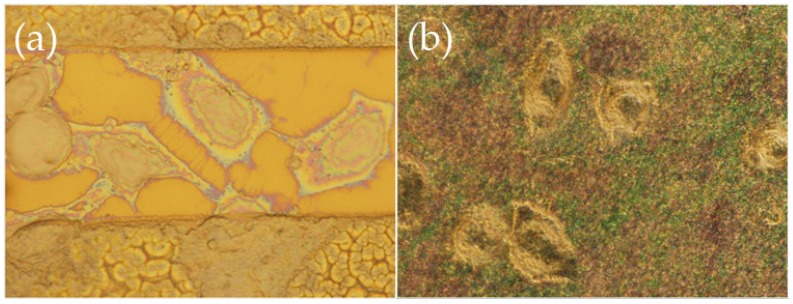
High magnification (×100) optical microscope images in dark field of HeLa cells after 4 days in culture on modified nitride samples that have adhered (**a**) on the nitride surface; and (**b**) onto the nanostructures. In (**a**), one can see the extensive filopodia network, while in (**b**) the more spherical shape of the cells can be discerned. The colorful specs are the tips of the ZnO nanostructures as seen in dark field.

When type “L” samples (large patterns and large flat areas) were employed as substrates, the preferential adhesion of the HeLa cells onto the flat areas was evident even from day 2 (Figure 4). On day 4, because of the very large cell population, numerous cells seem to be “forcibly” located onto the nanostructures, but they were fewer in number and their shape more spherical as was the case with type “S” templates. Again, after 4 days the cells appear rounder and do not have the usual oblong shape observed when cultured on flat surfaces (like conventional petri dishes or cover slips). It was very interesting to note that the cells that adhered onto the flat areas had a very large number of extended filopodia and were flat in shape (Figure 5). It seems that the flat areas provide a more favorable ground for the HeLa cells to create focal adhesions than the nanostructures.

As a conclusion, the HeLa cells tend to adhere preferentially onto flat areas when nitride layers are modified with ZnO nanostructures. In a recent publication by Migliorini *et al.* [61], it is suggested that the controlling factor in cellular adhesion might not be the nanostructuring itself but the mechanical properties of the substrata. This point is further analyzed in Section 3. Nonetheless, even in the case of modified nitride layers, the method seems to be very promising for culturing cells on predefined patterns.

### 2.3. HeLa Cultures on Emulated MEAs

**Figure 6:**
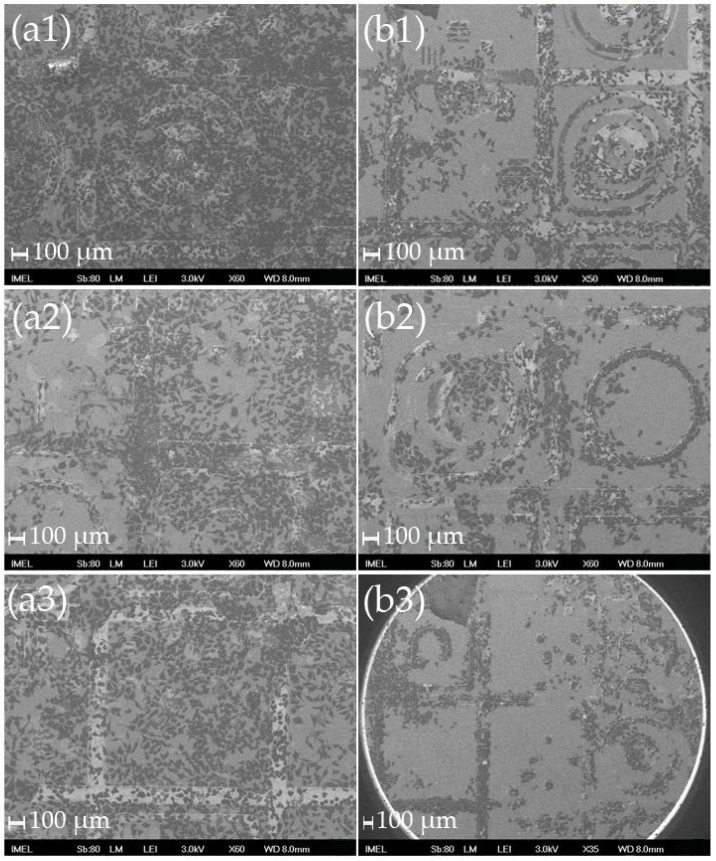
SEM images of HeLa cells on various patterns cultured on Al-MEAs after (**a**) 2 days; and (**b**) 4 days in culture where it can be seen that after day 2 the cells move preferentially to the nanostructured metallic patterns. Scale bar: 100 μm. (**a1**); (**a2**); and (**a3**) were obtained at several locations of the template after 2 days in culture. (**b1**); (**b2**); and (**b3**) were obtained at several locations of the template after 4 days in culture.

**Figure 7:**
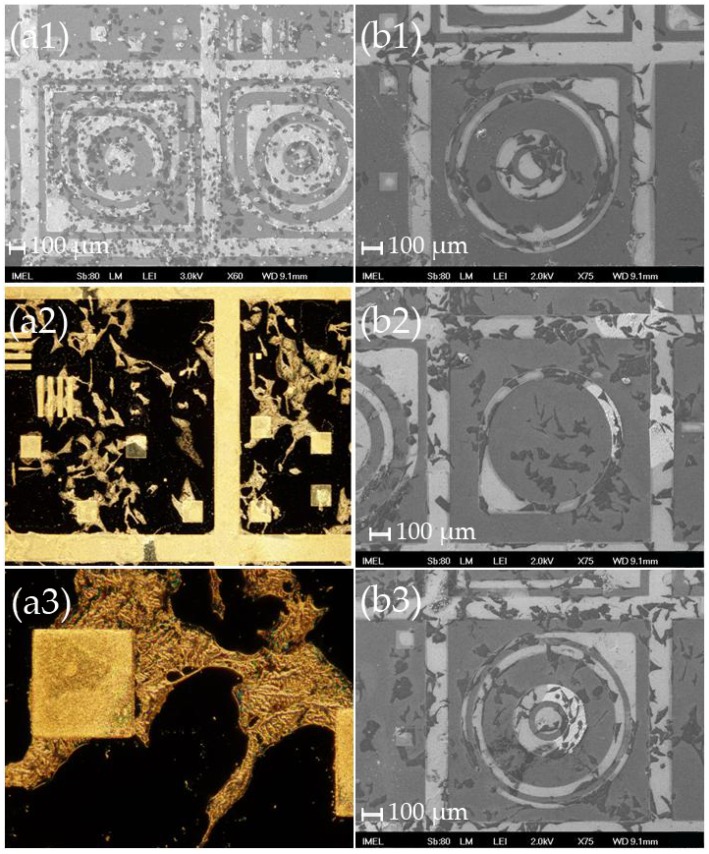
SEM and optical microscope images of HeLa cells cultured on Pt-MEAs after (**a**) 2 days (image a2: dark field, magnification × 10/ image a3: dark field, magnification × 50); and (**b**) 4 days in culture where it can be seen that after day 2 the cells move preferentially to the nanostructured metallic patterns. (**a1**); (**a2**); and (**a3**) were obtained at several locations of the template after 2 days in culture. (**b1**); (**b2**); and (**b3**) were obtained at several locations of the template after 4 days in culture.

Third types of sample realized with the proposed methodology were emulated microelectrode arrays (MEAs). In essence, Si 3”- and 4”-wafers were patterned with Al and Pt layers on top of which ZnO nanostructures were grown via the hydrothermal method (see Section 3 for fabrication details) emulating electrodes that could be locally modified with nanoarchitectures. Again, HeLa cells were cultured for up to 4 days and observed on day 2 and day 4, under exactly the same conditions as in Section 2.1 and Section 2.2. The results are summarized in Figure 6 and Figure 7.

On these templates, the HeLa cells—in stark contrast to modified Si and Si_3_N_4_ substrates—clearly formed networks on the predefined patterns seemingly guided by nanomechanical cues from the nanostructures. It was striking that until day 2 the cells just proliferated and adhered non-preferentially onto the flat surfaces and the nanostructured metallic patterns. However, between day 2 and 4, they migrated onto the nanostructured areas conforming totally to the patterns, as can be seen in Figure 6 and Figure 7 for the Al-MEAs and Pt-MEAs, respectively. It was also very interesting to notice that when there were lithographic imperfections the cells would still conform to the patterns and would not adhere to the flat surfaces (see for example Figure 6(b3)).

The results in the case of HeLa support the great potential of the method to be applied or controlled cellular cultures for HeLa cells. The cells are guided through the nanomechanical cues to specified patterns and a plethora of applications (ranging from single-cell observations and electrophysiology experiments to pharmacological studies) can be envisaged.

### 2.4. HeLa Cultures on Conventional Cover Slips

As a final type of substrate with hydrothermally-grown ZnO nanostructures, conventional cover slips were also chosen. The reason behind this option was that cover slips are commonly used in several optical methods for real-time recording of cells (like OWLS and in general video microscopy). Patterning of glass proved to be more challenging than anticipated and is analyzed in detail in Section 4. As a first step, cover slips uniformly covered with ZnO nanostructures were studied and compared to bare cover slips and Si wafers that were used as controls, since the results of Section 2.1 and Section 2.2 had shown that the ZnO nanostructures are not favorable to cell attachment. The initial cell population was 125 k/sample and the cells were examined after 1 and 2 days.

**Figure 8:**
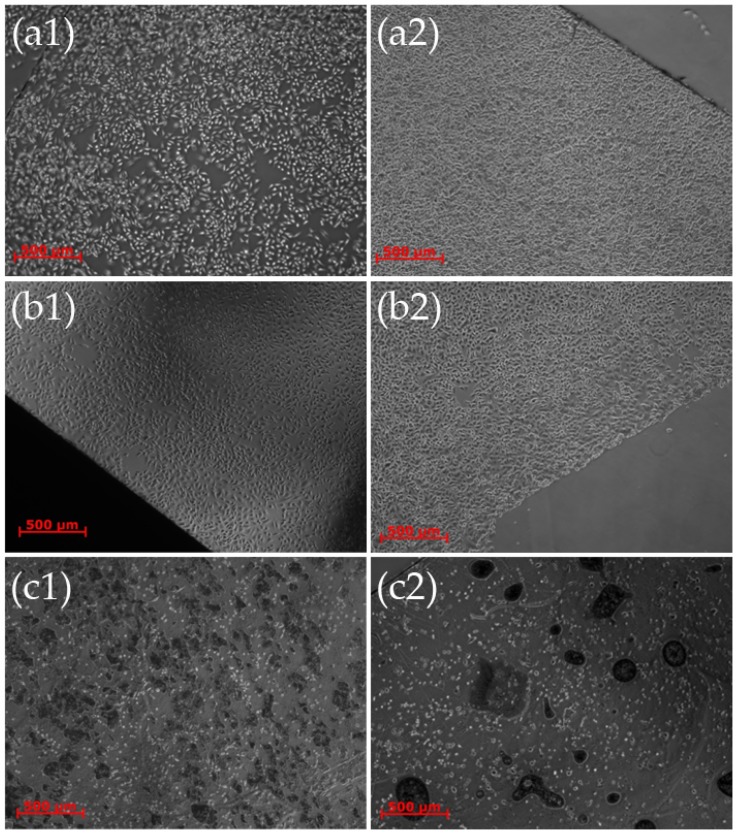
Optical microscope images (phase contrast images) of HeLa cells on (**a1)** control cover slips; (**b1**) bare Si; and (**c1**) cover slips covered with ZnO nanostructures after 24 h in culture; and (**a2**) control cover slips; (**b2**) bare Si; and (**c2**) cover slips covered with ZnO nanostructures after 48 h in culture. It is evident that the cells cannot adhere onto the ZnO nanostructures and undergo necrosis even from day 1.

Already at day 1, it was obvious that the HeLa cells could not easily adhere onto the ZnO nanostructures as evidenced by the decreased population compared to the controls as well as their spherical shape (Figure 8). On day 2, while the control samples were fully covered with cells, the modified cover slips had cells that seemed to have undergone necrosis. It was also observed that dead cells were floating on the culture medium in the petri dish.

**Figure 9:**
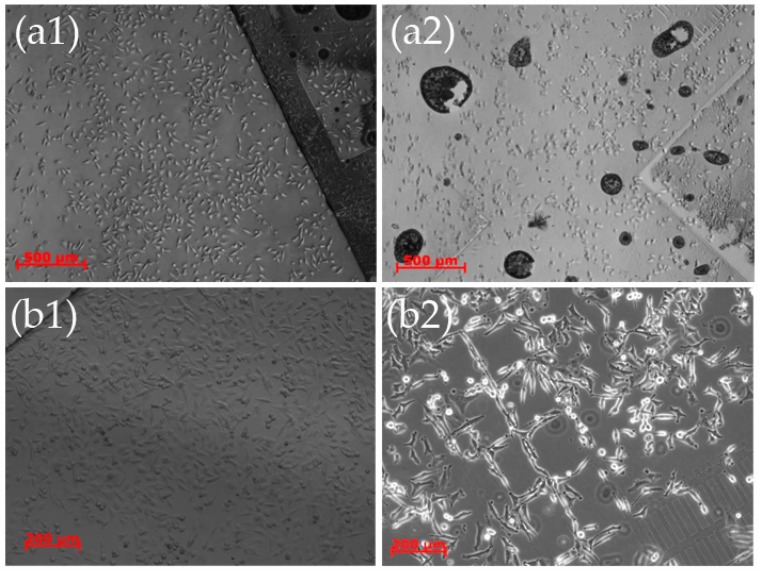
Optical microscope images (phase contrast images) of HeLa cells on (**a1**) a cover slip covered with ZnO nanostructures with small free (flat) patterns; and (**b1**) cover slip with ZnO nanostructures only on top of the small square lattice patterns after 24 h in culture; (**a2**) a cover slip covered with ZnO nanostructures with small free (flat) patterns; and (**b2**) cover slip with ZnO nanostructures only on top of the small square lattice patterns, after 48 h in culture. It is evident that the cells undergo necrosis after 24 h on the coverslips with the large patterns a1), while when the patterns are small (**b2**) there is total conformation of the cell somata to the borders of the patterns between day 1 and day 2.

As a next step, patterned cover slips were employed. Two types of patterning were used, one in which the cover slips were coated with ZnO nanostructures with only small flat patterns in between, and the exact negative *i.e.* bare cover slip with small patterns containing the nanorods (see Figure 13a and Figure 13b in Section 4). The cells were cultured for 48 h in total, but were checked under an optical microscope at 24 h, and then the culture was continued until 48 h, when the cells were fixed. At 24 h there was no evidence of preferred adhesion and the cells could be seen everywhere on both samples (Figure 9(a1,b1)). At 48 h though, as expected, the cells on the sample which in the most part was covered with ZnO, underwent necrosis (Figure 9(a2)), while the cells on the “negatively-patterned” cover slips migrated and conformed to the patterns (Figure 9(b2)).

These results show the great potential of nanopatterned cover slips for engineered cellular structures. The extremely low cost of the substrates and the equally low cost of the method make it a very promising route for a viable commercial product.

## 3. Discussion

For clarity reasons, and to facilitate the analysis and comparison of the results, the main observations have been summarized in Table 1. As a general conclusion, the proposed methodology seems very promising for the realization of cost-efficient templates for engineered cellular networks through the use of nanomechanical and nanotopographical cues onto a variety of substrates that can be applied to MEAs as well as optical biosensors. Still, one important parameter is the cell phenotype and it should be taken into account—as also seen in literature—that there is no universal nanotopographical feature that can regulate in a unique way the behavior of any cell type. Under this light, it is important to note beforehand that the results of this work may conflict with other reports in the literature or could potentially be entirely different if the templates were employed with other cell types.

Despite the fact that the cellular networks were not realized in all types of templates, there were strong indications that ZnO nanostructures can induce selective cellular adhesion and can guide HeLa cells onto predefined patterns and induce their adhesion on selected topographies. Given the low cost and the CMOS-compatibility of the method, the proposed methodology has a great potential to be used for viable products spanning from microelectrode arrays to modified optical sensors and really low-cost modified cover slips for real-time monitoring applications.

Coming to interpret the results per fabricated template, one should take into account that the cell guidance through nanomechanical cues is still an open issue in the literature, despite the large number of publications and studies. One difficulty in defining the controlling factors is that every publication employs different types of substrates, various micro/nanotopographical features, and most importantly different cell lines. In several cases, the findings of researchers may be contradictory, as was also the case in this work compared to [57] or from type of substrate to substrate within this work (e.g. modified Si and Si_3_N_4_ *versus* the emulated MEAs). Moreover, different cell lines react differently to the nanotopography and may have exactly the opposite responses. As Anselme *et al.* pointed out in their review paper “[…] all these observations illustrate the varying capability of cells of different phenotype to detect and react to nanotopographies and highlight the necessity of considering this parameter when a cellular model is chosen to study the influence of nanotopography on cell response. The generalization of results obtained with one cell type to another cell type is hazardous and must be avoided” [62]. 

Nonetheless, there is a general consensus in the literature that living cells are exquisitely sensitive to the local micro- and nanoscale topographic and biomolecular patterns constituting a complex and hierarchical adhesive ECM microenvironment in the three dimensions. A key controlling factor is the way focal adhesions may be formed and how their formation may be impacted by the nanotopgraphy and chemical topology at the nanometric scale, because this length scale is physiologically relevant due to the consistency of the nanopatterns with the sizes of many functional biomolecules and their complexes, including the fibers of ECM proteins, the components of basement membrane and focal adhesions [61,62,63,64].

Moving one order of magnitude upwards, from the 1–10 nm scale of ECM proteins to the sub-μm to μm-scale in cell structure, it has already been established in literature that filopodia play a pivotal role in the adhesion and functionality of cells. Since 1961 when they were observed for the first time in the living cell [65], and 1976 when their substrate—exploring function was suggested in the classic study of Albrecht-Buehler [66], various filopodial functions are now well established, such as directing the growth cones in neural networks [67], axon disorientation when filopodia formation is suppressed [68] or even blood clotting [69]. However, despite the numerous publications, the exact mechanisms remain elusive and the main difficulty arises—as it was previously mentioned—from the fact that every cell line reacts differently to the nanotopographical cues. Hence, it could be argued that the sensitivity of living cells to their environmental cues could be due to a dual effect of the nanotopography features both in the way focal adhesion form and in the nanosensory mechanisms underlying filopdia functions.

Taking the above into account, a general conclusion that could be derived from this work is that apart from the dependence of selective adhesion to the cell phenotype, the correlation of preferential cellular adhesion to nanotopographical features should be examined as a dynamic, time-dependent phenomenon and should not be merely treated as a static, non-reversible phenomenon. In other words, based on the observations and irrespective of the cell line examined, the selective adhesion is dynamic and the cells—which are in essence living organisms—continually react and adapt to their external micro/nano-environment. Hence, even though specific patterns and nanostructures were identified as substrates that can lead to selective adhesion and cellular network formation, time is a very important parameter and apart from the optimum geometry per cell line, the optimum culturing time must be identified as well.

This conclusion seems to be in agreement with the recent publication of Albuschies and Vogel [70]. Based on their study, the authors have developed a model describing the way the filopodia explore the topography of their environment and can adhere or not onto the substrates. Filopodia anchorage is a dynamic phenomenon and depends on the dynamics of contact angle formed towards flexible objects. It is this angle that defines whether the contact can be mechanically stabilized or peeled off. According to this zipping mechanism—as termed by the authors—topographical preference is not just an intrinsic and cell specific attribute. Instead, topographical preferences can change with time. Contact guidance might be a filopodia traction force-mediated peeling process. The cell is guided only in the direction where the geometrical constraints allow the filopodial contacts to mature by forming a maximum number of adhesive bonds. The contact angle formed between a filopodium and any object determines whether the contact can be stabilized or broken. Any synthetic of biological fiber free to swing around and align with filopodia will evoke a greatly different mechanosensation than bulk materials or interconnected fiber networks.

These conclusions are also in agreement with reference [71], where the time-dependence of selective adhesion was reported. The authors observed that cells that were initially adhered on specific nanotopographies migrated to “less-friendly” nanotopographies after a few days. It was also observed that the closer the “less-friendly” areas were to the preferred ones the faster they would fill with cells. In addition, a large number of cells would sit on the borderlines between the two areas early on in the cultures. The authors established that the cells that had selectively adhered onto the nanotopographies t enhanced anchorage synthesized ECM proteins, *i.e.* acted as “ECM sources”. ECM spreading, starting at the borderline between the two nanoscale topographies, increasingly masked the “unfavorable” nanotopography and enhanced cell adhesion. In other words, the initial cell adhesion and spreading were predominantly dependent on the nanotopography and the less-favorable areas could be “rescued” at least partially by ECM spread out of the adhered cells, which could then migrate in a step-by-step manner with the support of the ECM produced previously on the friendly nanotopography.

The behavior of both Neuro2A cells in [57] and HeLa cells reported in this work were analogous. Irrespective of which topography the cells preferentially adhere to (Neuro2A favor vertical nanorods, while HeLa prefer the flat surfaces or the nanostructured metallic layers) there is a clear time-dependence of the phenomenon. In particular, in the case of HeLa cells, the adhesion is preferential towards the flat surfaces (when the substrate is Si or Si_3_N_4_) and it seems that the cells tend to avoid the nanostructures. Noticeably enough, the more “Neuro2A-friendlier” a surface is, the less “HeLa-friendly” it becomes: the vertical nanorods are the areas that HeLa cells tend to avoid the most, while on the large-angled nanorods they have an “intermediate” behavior. Intense filopodia extension was observed only on the flat surfaces (Figure 5a). After day 2, the cells that have sat on the nanostructures lose their elongated shape and become rounder, indicative of necrosis (Figure 5b). This observation was further substantiated by the necrosis of HeLa cells cultured on the modified cover slips. Therefore, the particular nanotopography offered by ZnO nanorods does not promote the creation of focal adhesions for this cell line. In contrast, the HeLa behavior was exactly the opposite when cultured on the emulated MEAs. In this case, the HeLa cells adhered selectively onto the nanopatterned metallic layers conforming totally to the shape of the patterns. It must be underlined that the selective adhesion required 4 days to be realized as fully supporting the scenario of the cell dynamical behavior and rendering the culture duration as one of the controlling factors.

Of course, although the trends of adhesion selectivity are clearly visible, more work is required to reveal the underlying biological and biophysical mechanisms. Details such as the effects of ZnO surface nanostructuring, structure thickness and geometry on contact angle, and protein adsorption should be systematically investigated. These experiments are outside the scope of the present work, but using the developed methodologies could be carried out in a straightforward way, even using high throughput measuring formats. Further improvement of the performance and specificity could be obtained by combining the developed structures with cost-effective polymer or protein coatings containing one or more cell adhesion motifs.

In summary, the suggested methodology is extremely promising for the creation of engineered cellular networks through purely nanomechanical cues. One of the most important results of this study was a better understanding of the dynamics of selective cellular adhesion and the implication of time as a controlling factor that must be combined with nanomechanosensation. Future studies that are envisaged are the real-time monitoring of cell adhesion in order to further elucidate the phenomenon, and the application of the proposed templates to other cell lines and the realization of co-cultures. Finally, further studies are foreseen for the evolvement of the method into a technology that can be readily transferred to mass-production and the development of real-life viable products.

## 4. Materials and Methods 

### 4.1. Short Description of Sol-Gel/Hydrothermal Methodology

The suggested methodology has been widely used over the past ten years and was opted not only because of its versatility, but also for the following competitive advantages that it possesses over other fabrication methods: (a) it is facile, time-efficient and of very low cost, (b) clean-room conditions are not necessary, (c) it allows the control of the nanostructure morphology through simple key parameters (e.g. composition of the sol-gel, annealing temperature and duration, the nutrient solution concentration, pH and temperature, the growth duration *etc*.), (d) it is CMOS-compatible and can be integrated with standard microfabrication techniques, (e) it can be applied to a plethora of substrates, (f) it is non-hazardous and environmentally-friendly.

In essence, the method consists of two steps:

1. the deposition of a thin seeding (nucleation) layer that provides the necessary nucleation sites for the nanocrystal formation to commence. The deposition of the seeding layer is achieved via simple centrifugation of an appropriately selected sol-gel formed by the dissolution of zinc acetate dihydrate into an alcohol (ethanol or propanol), which may contain ethanolamine or triethylamine that promotes the formation of ZnO nanoparticles. It has been established that the seeding layer has a direct and critical effect on the structural properties of the ZnO nanostructures that are subsequently grown on top of it controlling the alignment, density, and morphological characteristics of the resulting structures. In other words, the seeding layer preparation conditions have a direct impact on the resulting structures and one can tailor them according to the targeted applications through several parameters like the number of spin-coatings, the annealing temperature and duration [29,72,73].
2. the hydrothermal growth step *per se*, during which the substrates are either immersed or floating in the nutrient solution most commonly constituted of (as in our case) zinc nitrate hexahydrate (ZnNO_3_.6H_2_O) and hexamethylentetramine (C_6_H_12_N_4_, HMTA) [28]. The nutrient solution is heated and the formation of the ZnO nanostructures is achieved through the following reactions taking place over the nucleation sites of the seeding layer:
C_6_H_12_N_4_ + 6H_2_O → 6HCHO + 4NH_3_ (1)
NH_3_ + H_2_O → NH_4_^+^ + OH*^−^* (2)
Zn^2+^ + 4OH^−^ → [Zn(OH)_4_]^2+^ (3)
[Zn(OH)_4_]^2+^ → ZnO_2_^2−^ + 2H_2_O (4)
Zn^2+^_surface_ + ZnO_2_^2−^_solution_ → 2ZnO (5)
O^2−^_surface_ + ZnO_2_^2−^_solution_ + H_2_O → ZnO + 4OH^−^ (6)

The role of HMTA has still not been fully elucidated. It is generally believed that it acts as a nonionic cyclic tertiary amine and thus serves as a Lewis acid base to metal ions and as a bidentate ligand capable of bridging two zinc ions in solution. The OH^−^ from the slow hydrolysis of HMTA assists the formation of the zinc hydroxide intermediate, which acts as a growth unit as shown in Formulas (2)–(4). The slow release of the hydroxyl groups may control the formation of the growing units, thereby exerting a profound effect on controlling the growth process of ZnO NRs. Coordination to the zinc of HMTA can also kinetically control the concentration of free zinc ion in solution and maintain the warmth of the reaction environment. Ammonia is provided by slowly decomposing HMTA with gradually increasing temperature. Zn^2+^ is known to coordinate in tetrahedral complexes. Zn^2+^ ions are stored by forming complex zinc [Zn(OH)_4_]^2+^. When the reaction temperature rapidly increases, HMTA is quickly hydrolyzed and produces a large amount of OH^−^ in a short period. Large amounts of ZnO nuclei form in the solution and aggregate together, thereby hindering the growth of ZnO NRs. Apart from the inherent rapid growth along the direction of the polar surfaces of the wurtzite ZnO crystal, the attachment of HMTA to the non-polar side facets also facilitate anisotropic growth in the [0001] direction. Thus, once the ZnO seeds are formed, the environment composed of Zn^2+^ and HMTA restrains the crosswise growth and guides the seeds to grow along the *c*-axis direction.

For each type of template fabricated in this work, appropriate sol-gels were selected and the various deposition conditions were tuned according to the substrate in order to achieve the desired ZnO nanostructure morphology. The hydrothermal growth step was kept the same for all templates and it was a 2-h growth at 87 °C in a 40 mM equimolar solution of ZnNO_3_∙6H_2_O and HMTA.

### 4.2. Fabrication of Si and Si_3_N_4_ Templates with Patterns of ZnO Nanostructures

**Figure 10:**
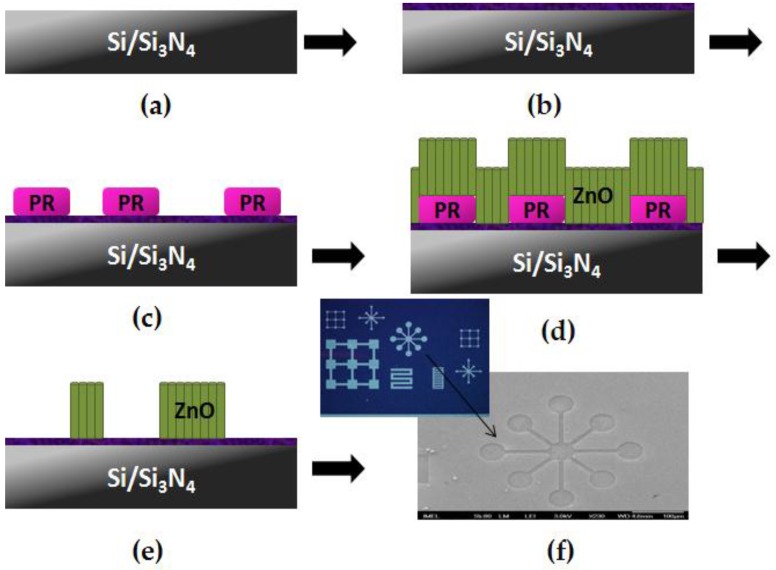
Schematic diagram of the process used to fabricate the patterned Si and Si_3_N_4_ templates (**a**) cleaning of substrates with organic solvents; (**b**) deposition of seeding layer; (**c**) optical lithography for the definition of patterns; (**d**) hydrothermal growth of ZnO nanostructures; (**e**) lift-off; (**f**) optical and SEM images of the templates.

**Figure 11:**
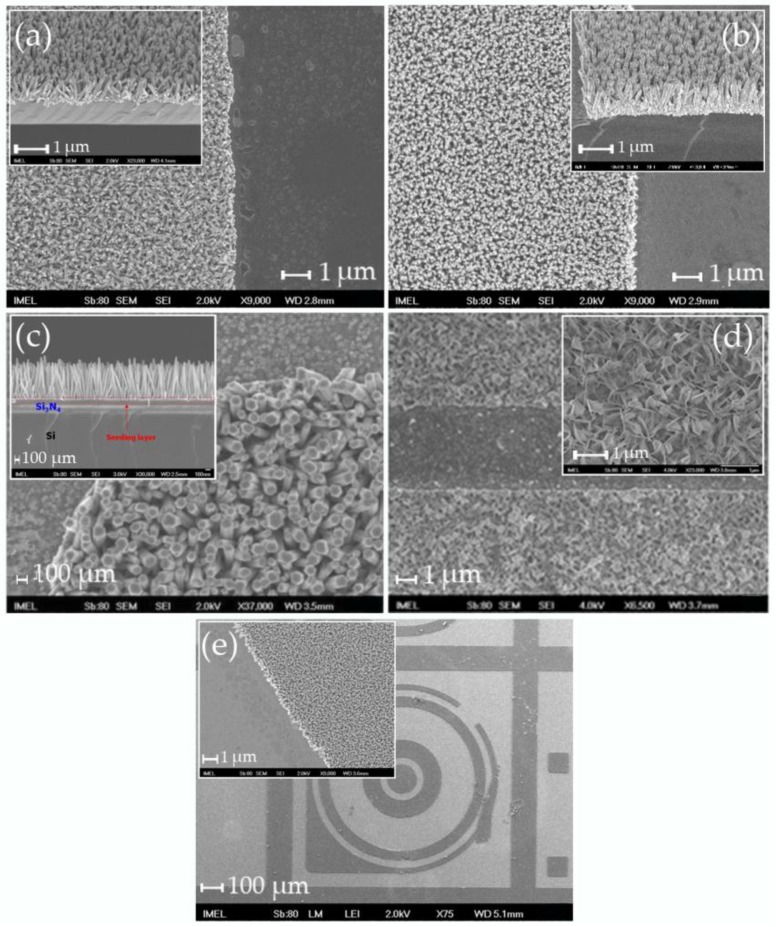
Typical SEM images of the templates used in this work for HeLa cell cultures: (**a**) Si templates with large angle ZnO nanorods; (**b**) Si templates with vertically-aligned, closed-packed ZnO nanorods; (**c**) Si_3_N_4_ template with vertically-aligned ZnO nanorods; (**d**) emulated MEA with Al patterns and ZnO nanoleaves; and (**e**) emulated MEA with Pt patterns and ZnO vertically-aligned nanorods. Insets: higher magnification images to exhibit in more detail the morphological characteristics of the ZnO nanostructures.

The process steps followed for the realization of Si and Si_3_N_4_ templates with patterns of ZnO nanostructures were identical and are schematically shown in Figure 10. Briefly, either bare Si or Si wafers covered with 100 nm-thick LP-CVD Si_3_N_4_ layer were cleaned with organic solvents and then spin-coated with a sol-gel for the formation of the seeding layer. The sol-gel consisted of 40 mM zinc acetate dihydrate (Zn(CH_3_COO)_2_.2H_2_O, Merck) dissolved in ethanol (C_2_H_6_O, Carlo Erba Reagents). The sol was prepared by vigorous stirring at 60 °C for 30 min and was employed after cooling at room temperature. The solution was spin-coated onto the substrates several times with 10 min drying steps in air in between. Subsequently, the samples were annealed at 500 °C in an oven in the presence of atmospheric air. Annealing for 1 h resulted in Si-templates with ZnO nanorods grown at large angles (Neuro2A-unfriendly templates), while annealing for 2 h resulted in vertical densely-packed ZnO nanorods (Neuro2a-friendly templates). The Si_3_N_4_ templates were all annealed for 2 h, since the scope of the study was to determine the effect of large *versus* small areas of nanostructures (see Section 2.2). After the seeding layer formation, optical lithography was performed in order to define the patterns over which the ZnO nanostructures were to be grown. The lithographic step was followed by the growth of the NWs at 87 °C in a 40 mM equimolar aqueous solution consisting of zinc nitrate hexahydrate (N_2_O_6_Zn.6H_2_O, Sigma-Aldrich) and hexamethylenetetramine-HMTA ((CH_2_)_6_N_4_, Panreac) for 2 h. The samples were finally cleaned with DI water in an ultra-sonic bath for 30 min. Typical images of the prepared templates are shown in Figure 11a–c.

### 4.3. Fabrication of Templates with Metallic Patterns Modified with ZnO Nanostructures (Emulated MEAs)

The fabrication process of the emulated MEAs was identical to the process used for the Si and Si_3_N_4_ templates with three additional steps before the formation of the seeding layer. After cleaning of the substrates, a first lithographic step was performed in order to define the patterns of the metallic layers, followed by e-beam evaporation of 1000 Å of Al or thermal evaporation of 500 Å of Pt and then a lift-off step. After the definition of the metallic patterns, the seeding layer was formed as described in Section 4.2. The second lithographic step employed the same mask with care taken to align the patterns on top of each other. As in the previous case, after the lithography, the hydrothermal growth was realized followed by a final lift-off step and cleaning with DI water. The hydrothermal growth conditions were kept the same. Typical images of the emulated MEAS are shown in Figure 11d–e. It is worth noting that ZnO nanostructures grown on Al layers do not assume the “conventional” rod-like shape, but instead obtain a quite distinct form of nanoleaves or nanosheets (see Figure 11d). 

### 4.4. Fabrication of Cover Slip Templates with Patterns of ZnO Nanostructures

As far as the modification of conventional cover slips is concerned, the process proved to be more complicated than anticipated. On the one hand, it was challenging to pinpoint the exact conditions that could lead to uniform seeding layer formation, and on the other hand it was equally challenging to find the exact conditions in which the seeding layer would withstand the lithographic step.

After extensive trials, it became obvious that the most suitable sol-gel for glass substrates is a 500 mM zinc acetate dihydrate solution in propanol with the addition of ethanolamine. In detail, zinc acetate dihydrate is dissolved under vigorous stirring at 60 °C over a hot plate. After homogenization of the solution, which is milky in color since the solution is supersaturated, ethanolamine is added. Upon addition of ethanolamine, the solution becomes clear. Stirring is continued for another 30 min and then the sol-gel is left to cool to room temperature and may be used after 12 h.

Moreover, it was established that the strategies for spin-coating and annealing employed for Si and Si_3_N_4_ substrates could not be successfully applied to glass. For example annealing at 500 °C is not feasible, because glass cannot withstand such temperatures. Therefore, the annealing temperatures should range between 120 °C (minimum temperature for the decomposition of the acetic radical from zinc acetate and the uptake of atmospheric oxygen for ZnO formation) and 350 °C so as the glass substrates do not crack Finally, it was proven that HMDS (promoter) must be used prior to sol-gel spin-coating to enhance adhesion. Therefore, the seeding layer preparation for the cover slips was transformed as follows: first HMDS is spin-coated onto the samples followed by a short soft bake at 90 °C for 30 s. Then the sol-gel is spin coated at low revolutions followed by a new coating at higher velocities. Finally, the samples are annealed at 350 °C for 2 h on a hot plate in ambient conditions.

**Figure 12:**
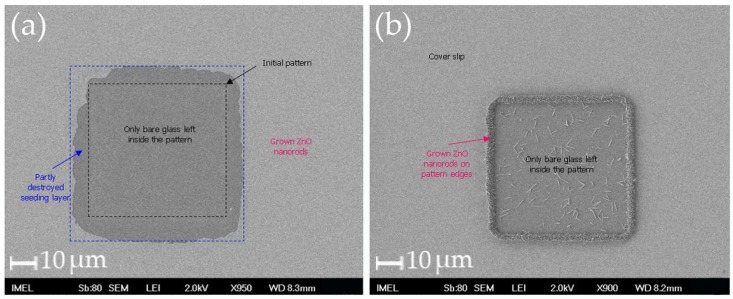
SEM images of cover slips after lithography under various conditions where the removal of the seeding layer and the degradation of the patterns can be seen. The intention was to grow ZnO nanorods only within the square patterns. (**a**) slight overexposure and overdevelopment were used; the seeding layer is completely removed after being degraded by the UV exposure and the developer which acts as an etchant has sipped under the photoresist in a way similar to creating undercuts in wet etching techniques (**b**) optimum exposure time and underdevelopment (the seeding layer “survives” only under the borderlines of the photoresist where the photoresist is slightly thicker, while the developer which acts as an etchant does not have the time to sip through. As a result ZnO nanorods grew only at the circumference of the pattern).

**Figure 13:**
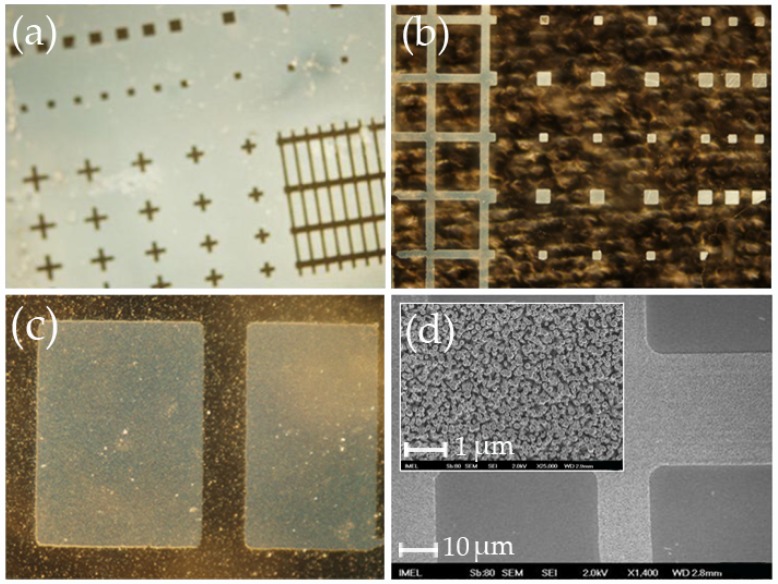
(**a**)–(**c**) Typical optical microscope images (dark field) of cover slips modified with ZnO nanostructures. Sample in (**b**) is the negative of sample in (**a**) showing the versatility of the method. (**d**) SEM image that shows the morphology of the ZnO nanorods. [Magnification in (**a**) and (**b**): ×10; Magnification in (**d**): ×50; Scale bar of inset in (**d**): 1 μm].

After defining the proper condition for the seeding layer formation on blank samples, the next step included the lithographic patterning of the cover slips. However, when standard optical lithography was applied, the patterns were degraded and in the majority of cases the seeding layer was totally removed (see Figure 12). The degradation and removal of the seeding layer is most probably due to the degradation of the layer during the exposure step, since both glass and ZnO absorb in the UV. It also became clear after several combinations of under/over-exposure and under/over-development steps that the developer acts as an etchant for the seeding layer as shown in Figure 12. The fact that the lithographic step destroys the seeding layer was used in an alternative way to modify the cover slips. Instead of the conventional lift-off step, the lithographic step itself was employed to define the patterns: after removing the seeding layer with the lithographic step, the photoresist was stripped off and the samples were immersed into the growth solution, since ZnO cannot grow onto bare glass. Typical images can be seen in Figure 13, where both negative and positive patterns of the same mask were fabricated.

### 4.5. Solubility Studies on ZnO Nanostructures

Several studies have raised concern about possible cytotoxicity of Zn ions or ZnO nanoparticles (e.g. [74,75]). Therefore, in order to eliminate the dissolution of ZnO nanostructures as a factor for the observed cell necrosis or difficulty to adhere onto the nanopatterns, solubility studies of the templates were conducted prior to cell culturing. We investigated their solubility in solutions with acidic, basic, and neutral pH. The plates were soaked in hydrogen chloride (pH = 2), in potassium hydroxide (pH = 12), and in Milli-Q “ultrapure”water (pH is around 6) for one hour. Images were taken before and after the treatment by scanning electron microsopy (SEM).

**Figure 14:**
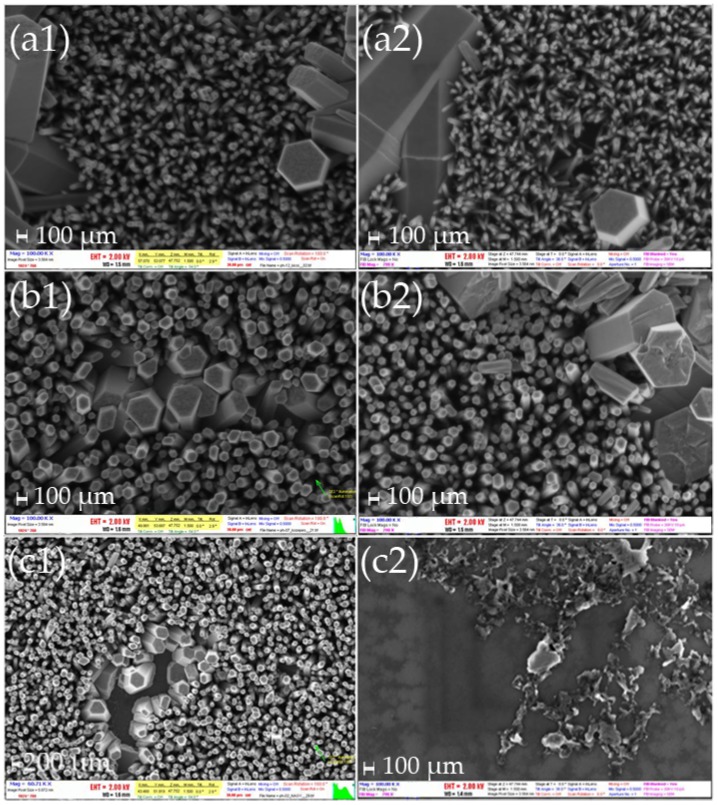
SEM images of the ZnO nanostructures before (left column) and after being immersed for 1 h (right column) in (**a**) an alkaline solution with pH = 12, (**b**) neutral solution with pH 6, and (**c**) an acidic solution with pH = 2. Images (**a1**); (**b1**); and (**c1**) were obtained before immersion in the solutions; (**a2**); (**b2**); and (**c2**) were obtained after 1 h in the solutions.

As shown in Figure 14, alkaline and neutral (pH = 12 and 6) liquids and solutions do not affect the nanoscale morphologies of the structures (Figure 14a,b, respectively). The nanowires remained on the surface and they were intact. However, the acidic solution (pH = 2) strongly affected the nanostructures by totally dissolving the nanowires (Figure 14c). Therefore, it was established that the ZnO nanostructures do not disintegrate in the cell-culture medium and the limited cell adhesion and viability are mostly related to the nanotopographical cues.

### 4.6. HeLa Cell Cultures

Human cervix carcinoma HeLa cells were obtained from the European Collection of Cell Cultures (ECACC). The cells were maintained in Dulbecco’s Modified Eagle’s Medium (DMEM, Sigma-Aldrich, Budapest, Hungary), supplemented with 10% fetal bovine serum (Biowest, Nuaillé France), 4 mM L-glutamine (Sigma-Aldrich, Hungary), 100 U/mL penicillin, 100 µg/mL streptomycin solution (Sigma-Aldrich, Budapest, Hungary) and 0.25 µg/mL amphotericin B (Sigma-Aldrich, Budapest , Hungary) in a humidified atmosphere containing 5% CO_2_ at 37 °C. On reaching 80% confluence, cells were detached every 2–4 days using 0.05% (w/v) trypsin, 0.02% (w/v) EDTA solution and not used beyond passage 20.

## Figures and Tables

**Table 1:** Summary of the observations for the various templates according to the material used as a substrate.

ZnO Nanostructures on Patterned Si	ZnO Nanostructures on Patterned Si_3_N_4_	ZnO Nanostructures on Patterned Al	ZnO Nanostructures on Patterned Pt	ZnO Nanostructures on Patterned Cover Slips
***Substrates with small patterns:*** Opposite behavior to Neuro2A increased population onto nanorods at large angles decreased population onto vertical nanorods The flat areas too small to accommodate cell somata/not possible to determine selective adhesion	***Substrates with small patterns:*** Cells tend to remain on flat areas by squeezing the somata inside the flat areas Flat areas too small to accommodate the cell somata/ not possible to determine selective adhesion	***2 days:*** No preferential adhesion	***2 days:*** Cells are more spherical and smaller compared to the Al-MEAs Conformation to the patterns at the borderlines- no clear preference	***Cover slips uniformly covered with ZnO nanostructures***: Necrosis since day 1
***Substrates with large patterns and large flat areas:*** Selective adhesion onto flat surfaces and avoidance of nanostructured areas Cell spreading and conformation to the patterns at the borderlines	***Substrates with large patterns and large flat areas*** Selective adhesion onto the flat surfaces and avoidance of nanostructured areas Cell spreading and conformation to the patterns at the borderlines	***4 days:*** Clear migration and selective adhesion of cells onto nanopatterned metallic layers—almost all cells adhere onto the nanopatterns	***4 days:*** Clear migration and selective adhesion of the cells onto the nanopatterned metallic layers—almost all cells adhere onto the nanopatterns Cells obtain their “conventional” elongated shape	***Cover slips with large ZnO nanostructure patterns***: Cell necrosis after day 1 ***Cover slips with small ZnO nanostructure patterns:*** Clear migration and selective adhesion of the cells onto the nanopatterned areas after day 1
